# The Effect of Germanium-Loaded Hydroxyapatite Biomaterials on Bone Marrow Mesenchymal Stem Cells Growth

**DOI:** 10.3390/cells11192993

**Published:** 2022-09-26

**Authors:** Jeevithan Elango, Rodion Bushin, Artiom Lijnev, Piedad N. De Aza, Carlos Pérez-Albacete Martínez, José Manuel Granero Marín, Ana Belen Hernandez, Luis Ramón Meseguer Olmo, José Eduardo Maté Sánchez De Val

**Affiliations:** 1 Department of Biomaterials Engineering, Faculty of Health Sciences, UCAM-Universidad Católica San Antonio de Murcia, Guadalupe, 30107 Murcia, Spain; 2 Center of Molecular Medicine and Diagnostics (COMManD), Department of Biochemistry, Saveetha Dental College and Hospitals, Saveetha Institute of Medical and Technical Sciences, Saveetha University, Chennai 600 077, India; 3 Instituto de Bioingeniería, Universidad Miguel Hernández, Avda. Ferrocarril s/n, Elche, 03202 Alicante, Spain; 4 Oral Surgery and Oral Implantology Department, UCAM-Universidad Católica San Antonio de Murcia, 30107 Murcia, Spain; 5 Department of Implant Dentistry, Faculty of Medicine and Dentistry, UCAM-Universidad Católica San Antonio de Murcia, 30107 Murcia, Spain; 6 Tissue Regeneration and Repair Group, Biomaterials and Tissue Engineering, Faculty of Health Sciences, UCAM-Universidad Católica San Antonio de Murcia, Guadalupe, 30107 Murcia, Spain

**Keywords:** hydroxyapatite, germanium, drug release, mesenchymal stem cells, protein adhesion

## Abstract

Hydroxyapatite (HA) is a hard mineral component of mineralized tissues, mainly composed of calcium and phosphate. Due to its bioavailability, HA is potentially used for the repair and regeneration of mineralized tissues. For this purpose, the properties of HA are significantly improved by adding natural and synthetic materials. In this sense, the germanium (Ge) mineral was loaded in HA biomaterial by cold isostatic pressure for the first time and characterization and biocompatibility using bone marrow mesenchymal stem cells (BM-MSCs) were investigated. The addition of Ge at 5% improved the solubility (3.32%), stiffness (18.34 MPa), water holding (31.27%) and biodegradation (21.87%) properties of HA, compared to control. Compared to all composite biomaterials, the drug-releasing behavior of HA-3% Ge was higher at pH 1 and 3 and the maximum drug release was obtained at pH 7 and 9 with HA-5% Ge biomaterials. Among the different mediums tested, the DMEM-medium showed a higher drug release rate, especially at 60 min. HA-Ge biomaterials showed better protein adhesion and apatite layer formation, which ultimately proves the compatibility in BM-MSCs culture. Except for higher concentrations of HA (5 and 10 mg/mL), the different concentrations of Ge and HA and wells coated with 1% of HA-1% Ge had higher BM-MSCs growth than control. All these findings concluded that the fabricated HA biomaterials loaded with Ge could be the potential biomaterial for culturing mammalian cells towards mineralized tissue repair and regeneration.

## 1. Introduction

Natural minerals have continuously been used in biomedical applications due to their unique physicochemical and biocompatible properties. For instance, various natural-derived minerals and metals are widely used in tissue regeneration applications. In this aspect, hydroxyapatite (HA) is a well know ceramic mineral, primarily composed of phosphate and calcium that is extensively used in bone and dental tissue regeneration and repair. For this purpose, many researchers fabricated different types of HA materials in the form of the scaffold, graft, 3D matrix and hydrogel [1,2,3]. More specifically, the biological behavior of HA has been improved by fabricating with other bio(materials), such as silk fibroin [4,5], cellulose [6], alginate [7], polylactic acid [8], Poly(𝜀-caprolactone) [9], collagen [10,11,12], polymethylmethacrylate [13] and gelatin/chitosan/polyvinyl alcohol [14], for better use in bone and dental tissue regeneration. Recently, several initiatives for the extraction methods are started to obtain HA from biowaste resources, such as fish bone, scale [15,16], rice husk and chicken eggshells [17,18,19].

In addition to HA, Germanium (Ge) is a naturally derived metalloid that structurally and physicochemically resembles diamond and silicon, respectively. Due to their excellent semiconductive properties, Ge is the first element used for the construction of transistors and semiconductors and has been triggering great interest in the field of biomedicine. The pharmacological studies already proved the antimicrobial, antimutagenic, antitumor, antiviral, immunomodulating and erythropoietic effects of Ge [20]. Though Ge has been commercially available for several decades, they recently got attracted to the treatment of certain diseases. For instance, organogermanium compounds have been used to treat AIDS, cancer [21], hemocompatibility [22], chronic hepatitis [23] and hematopoiesis [24] and to stimulate the immune system [25]. The biological activity of Ge depends on the chemical form and in most cases the Ge is used in the form of Ge132-beta-carboxyethylgermanium sesquioxide ((GeCH_2_CH_2_COOH)_2_O_3_), spirogermanium and germanium-lactate-citrate. Interestingly, a recent study demonstrated the low acute and chronic adverse effects of inorganic germanium dioxide [26]. Due to their excellent antioxidants, biocompatibility, immunostimulatory, low toxic (except GeH_4_, GeO_2_ and GeCl_4_) [21] and regenerative effect, several researchers have used Ge as a potential drug loaded with other polymers to explore the applicability in tissue regeneration. In a recent study, Ge was incorporated as a biologically active substance with graphene films to stabilize the physicochemical properties, electrical conductivity and surface properties. Their findings disclosed that Ge incorporated graphene had enhanced biological activity through upregulating osteogenic markers gene expression in mesenchymal stem cells (MSC) [27]. The beneficial effects of Ge on MSCs were reported recently with or without incorporating other polymers [27,28]. So far Ge has been incorporated with different polymer systems, such as hydrophilic N-doped ultrathin graphite scaffold for advanced electrodes [29], silicon cluster [30], metal ions (Cr^+^ and Ni^+^) for the growth of small microorganisms and biofilms [31], hollow carbon spheres for lithium-ion batteries [32], gold ion implantation for bioactive surfaces [33] and hyaluronic acid-graft-dopamine hydrogel for spinal cord injury repair [34].

Though, HA and Ge have been fabricated with various synthetic and natural materials, the combined effect of Ge and HA has not yet been explored. Ge element as a good conductor and HA as a major component of bones (55%) are promising materials for tissue regeneration and self-healing ability by transmitting signals in biological cells. Therefore, it is necessary to understand the potential effect of Ge combined with HA in biomedical applications. As an initiative, this study aimed to optimize the appropriate composition of HA and Ge for the biocompatibility of bone marrow mesenchymal stem cells (BM-MSCs) growth. For this purpose, solid-state-synthesized HA was isostatically cold pressed with Ge for fabricating biomaterials and their drug-releasing, protein adhesion, mineral deposition and biocompatibility for BM-MSCs growth were investigated.

## 2. Materials and Methods

### 2.1. Raw Materials and Composition

The raw materials for the present study were commercial Germanium, 2-Carboxyethyl germanium sesquioxide (GeCH_2_CH_2_COOH)_2_O_3_ (Cat No. 21030164 Japan algae Co., Ltd., Tokyo, Japan) and synthesized HA.

### 2.2. Hydroxyapatite Synthesis

HA was synthesized by a solid-state reaction from a stoichiometric mixture of calcium hydrogen phosphate anhydrous (CaHPO_4_, Sigma, St. Louis, MO, USA) and calcium carbonate (CaCO_3_, Sigma, St. Louis, MO, USA) with an average particle size of <15 µm and a Ca/P ratio of 1.72. The mixture of CaHPO_4_ and CaCO_3_ was heated in a platinum crucible to 1200 °C for 6 h at a heating rate of 10 °C/min followed by a cooling rate of 6.5 °C/min until room temperature. The obtained material was ground to an average particle size of 4.8 μm (Mastersizer APA 2000 E Ver. 5.20, Serial Number: MAL1013724, Malvern Instruments Ltd., Malvern, UK).

### 2.3. Fabrication of HA-Ge Composite

The respective components were weighed-out and thoroughly dry mixed in a mixing miller with PSZ-zirconia balls. After the milling process, the powder mixture was cold iso-statically pressed at 200 MPa. The pressure was maintained for 30 min, after which it was slowly depressurized to 1 atmosphere. In total, four types of samples were fabricated: control (100% HA), HA-1% Ge (99% HA with 1% Ge), HA-3% Ge (99% HA with 3% Ge) and HA-5% Ge (99% HA with 5% Ge).

### 2.4. Composite Characterization

The materials were characterized by X-ray diffractometer (XRD) (Bruker-AXS D8Advance, Karlsruhe, Germany) analyses of the raw materials and the ceramic composites were performed to determine crystallography and phase identification using Cu-Ka radiation at 40 kV and 30 mA. The scans were performed with 2 θ values varying from 5° to 65° at a rate of 0.05°/min. The average particle size of HA was estimated by particle size distribution test using Granulometry (Mastersizer APA 2000 E). HA-Ge composite morphology was done by scanning electron microscopy in a SEM-Hitachi S-3500N device with an energy-dispersive X-Ray spectroscopy (EDS-INCA, Oxford Instruments Analytical, Wycombe, UK).

### 2.5. Mechanical Properties

The compression strength of HA-Ge biomaterial was determined by using a texture analyzer (Model: Brookfield, Model CT3 50K, Middleborough, MA, USA). Briefly, the biomaterials with 10 mm × 9 mm were placed on the sample holder and potential force was applied using a plunger attached to the instruments and the compression strength of HA-Ge was measured by calculating stress–strain curves.

### 2.6. Solubility

The solubility of HA-Ge biomaterial was evaluated in different mediums, such as water, PBS and DMEM culture mediums. The initial weight (W0) of samples after drying at 60 °C for 5 h was measured and the samples were immersed in the above medium for 7 days. Then, the samples were dried at 60 °C for 5 h and the final weights (W7) were measured. The percentage of sample solubility was measured by the following formula. All the sample weights were normalized to 1 g for comparison.

Ws = ((W0 − W7)/W0) × 100, where Ws-water solubility percentage, W0 and W7-initial and final weight of samples, respectively.

### 2.7. Water Absorption Rate

The water absorption rate of HA-Ge biomaterial was measured by following the earlier method [35]. Briefly, the samples were dried at 37 °C for 36 h and weighed before immersion in distilled water. The samples were immersed in distilled water for 2 h at 37 °C and weighed the wet weight after gently removing the surface-bound excess water by filter paper. The water absorption rate was calculated according to the below equation.

WAR = (Ww − Wd)/Wd × 100%, where Ww and Wd are wet and dry weights of samples, respectively.

### 2.8. Swelling Rate

The swelling rate of HA-Ge biomaterial was measured by calculating the biomaterials’ surface area before and after immersing the biomaterials in phosphate-buffered saline (PBS) (Labclinics, Barcelona, Spain). The percentage of swelling rate was calculated using the formula:

SR = ((Aa − Ab)/Ab) × 100, where Ab and Aa are the area of biomaterials before and after immersion, respectively.

### 2.9. In-Vitro Degradation

The rate of degradation of HA-Ge biomaterial was measured by following the earlier method [36] with slight modification. Briefly, the biomaterial samples were immersed in 3 mL of Trypsin (Labclinics, Barcelona, Spain) and the enzymatic digestion was initiated by incubating at 37 °C. The initial weight of the sample (Wi) was measured after immersion in Trypsin solution and the weight of degraded samples (Wd) was measured periodically to calculate the percentage of degradation.

Degradation rate = ((Wi − Wd)/Wi) × 100

### 2.10. Water Angle Contact

The wettability of the sample was measured by water contact angles using drop shape analysis at room temperature. Briefly, 5 µL of deionized water was dropped on the surface of samples and the contact angles were calculated on both sides of the drop. Prior to the experiment, the samples were acclimatized at room temperature (with a RH of 60%) for moisture equilibrium.

### 2.11. Protein Adsorption Ability

The ability of biomaterials to absorb functional proteins on their surface was evaluated by Coomassie brilliant blue (CBB) test by following the earlier method [37]. Briefly, the biomaterials were treated with concentrated ethanol overnight to modify the surface characterization and both ethanol-treated and untreated biomaterials were incubated in 1 mL fetal bovine serum (Lot No. 2445724RP, Gibco, Carlsbad, CA, USA) at 37 °C overnight, washed with PBS thrice, fixed with 4% paraformaldehyde for 15 min and the surface absorbed proteins were stained with CBB G-250 (0.25% CBB G-250 in methanol/water/acetic acid, 40:50:10 volume ratio mixture) for 30 min. After staining, the biomaterials were de-stained with a methanol/water/acetic acid mixture overnight. Then, the samples were crushed in PBS and supernatant was measured at 590 nm. The amount of surface absorbed protein on HA-Ge biomaterial was calculated based on the absorption curve of standard protein with different concentrations, as reported earlier [38].

### 2.12. Bioactivity

The efficiency of HA-Ge in mineralization was evaluated by the method of Kokubo et al. using simulated body fluid (SBF) [39]. The SBF (pH 7.4) was prepared exactly following the same steps and recipes, as recommended by Kokubo et al. [39]. The samples were incubated vertically in 50 mL falcon tubes with SBF for 28 days at 37 °C with changing the medium every 3 days once. Then, the samples were gently washed with distilled water three times and stained with an alizarin red stain to visualize the mineral deposition on the surface. The amount of staining rate was optically measured by UV-Vis spectrophotometry (Bio Tek Instruments Inc., Winooski, VT, USA) at 450 nm by subtracting the control value (samples without SBF treatment). For quantification, the alizarin red stain was dissolved in a mixture of methanol (20%) and acetic acid (10%) in water for 20 min [40].

### 2.13. Drug Release Behavior

The drug releasing pattern of HA-Ge biomaterial was done by varying parameters such as pH and solvents. Briefly, the biomaterials were immersed in a solution with different pHs 1, 3, 5, 7 and 9, respectively, for 5, 20, 40, 60, 80 and 100 min. Moreover, the drug-releasing pattern of biomaterial was determined in a different solvent system, such as PBS, DMEM culture medium and simulated body fluid for 5, 15, 30, 45, 60, 75 and 90 min. The simulated body fluid was prepared as per the instructions of Kokubo et al. [39]. At each time point, the release rate of germanium in the supernatant was measured at 215 nm (as determined by the spectral absorption of germanium powder, Supplementary Figure S1). The amount of released germanium in the supernatant was calculated from the standard curve (Supplementary Figure S2) obtained with different concentrations of germanium 0.3125, 0.625, 1.25, 2.5, 5 and 10 mg/mL, respectively.

### 2.14. In-Vitro Cell Culture

The biocompatibility of HA-Ge biomaterials was assessed using in vitro cell culture of BM-MSCs. The cells were isolated from three healthy patients scheduled for elective orthopedic surgery in accordance with the criteria established by the International Society for Cell Therapy. The surface characterization of cells using CD73, CD90, CD105, CD44 and CD14/19/34/45 markers was already reported in our previous work [41].

BM-MSCs were cultured as per the standard cell culture protocol using DMEM medium supplemented with 10% FBS and 1% antibiotics (P/S) at 37 °C in a 5% CO_2_ incubator. Firstly, the cells were treated with germanium (0.001, 0.01, 0.25, 0.5, 1, 5 and 10 mg/mL) and hydroxyapatite (0.01, 0.1, 0.25, 0.5, 1, 5 and 10 mg/mL) in a different concentrations at 37 °C for 7 d in a 48 well plates. At the same time, the cells were cultured with HA-Ge disc or composites (1 mg/mL) coating in 24 well plates at 37 °C for 7 d. The proliferation and morphology of BM-MSCs were evaluated as per the standard protocol using automated Invitrogen cell counter (Countess 3 FL, Thermo Fisher Scientific, Waltham, MA, USA) and staining (H&E and FITC-DAPI) methods, respectively.

### 2.15. Cell Loading Density

The BM-MSCs at a cell density of 5 × 10^4^ were seeded on top of the biomaterials in order to evaluate the maximum amount of cell-loading capacity of HA-Ge composite. After 6 h incubation in a CO_2_ incubator at 37 °C, the biomaterials were moved to a new well and the adherent cells were counted after trypsinization using an automated cell counter. The percentage values were calculated from the seeding density of BM-MSCs.

### 2.16. Statistical Analysis

All the experiments were repeated at least three times and data were represented as mean and standard deviation. The statistical analysis was done using ANOVA using GraphPrism 9.0.1 (GraphPad Software Inc., San Diego, CA, USA). A *p*-value less than 0.05 was considered statistical significance.

## 3. Results

### 3.1. Characterization

**Figure 1:**
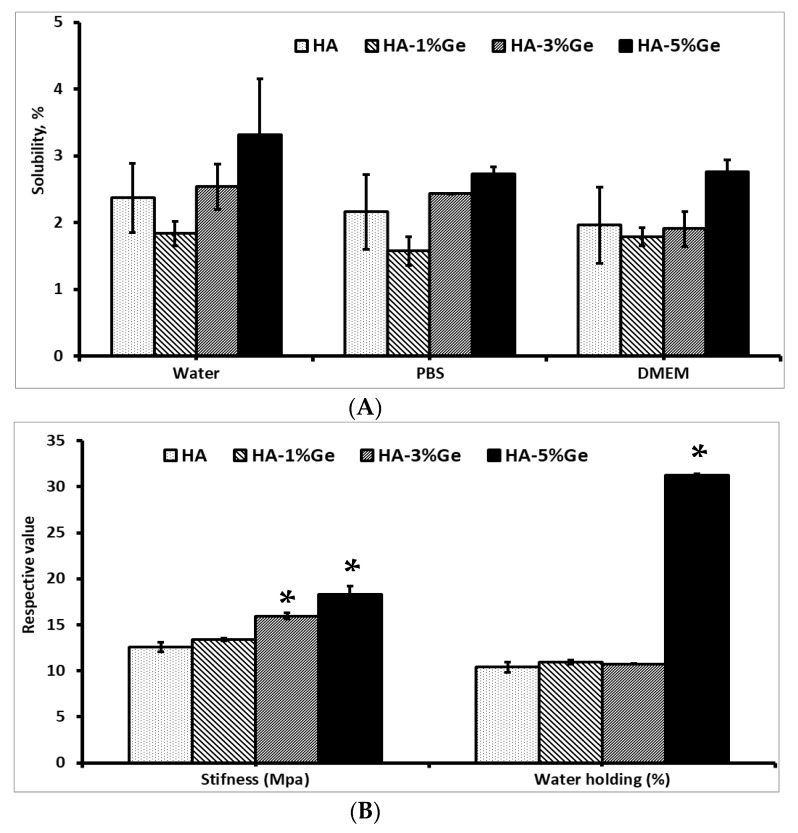

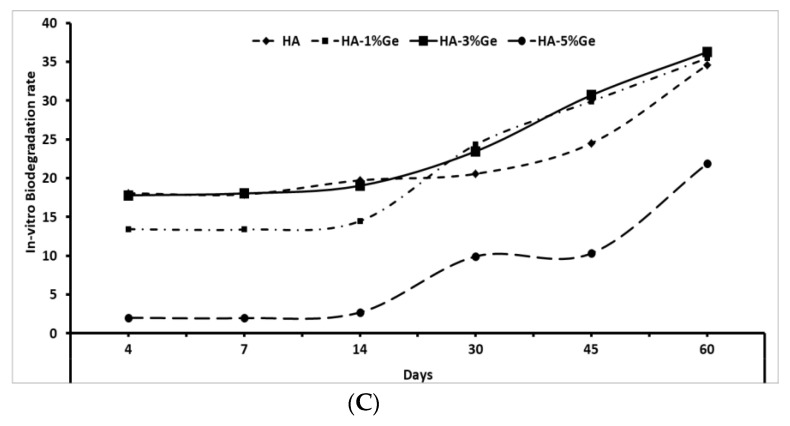
Solubility (**A**), stiffness, water holding (**B**) and in vitro biodegradation (**C**) of HA-Ge biomaterials. HA-control, HA 1% Ge: HA with 1% germanium, HA 3% Ge: HA with 3% germanium, and HA 5% Ge: HA with 5% germanium. * denotes statistical significance, *p* < 0.05 vs. HA.

The characteristics, such as solubility, stiffness, water holding and in vitro degradation of HA-Ge samples, are shown in Figure 1. The solubility of the sample was tested in three different mediums, such as water, PBS and DMEM, and the results showed that no significant changes were observed between control (HA sample) and test samples (except HA-5% Ge sample) in water. On the contrary, the solubility was slightly decreased in HA-1% Ge, however, increased in HA-5% Ge sample incubated in water, PBS and DMEM, respectively. Notably, the higher concentration of Ge-incorporated HA sample was more soluble in water, compared to PBS or DMEM (*p* > 0.05). Notably, there was no significant change observed in the swelling properties of HA and HA-Ge biomaterials throughout the study. Regarding the mechanical property, the addition of Ge up to 3% did not contribute any significant changes in stiffness though slightly increased the stiffness and 5% Ge could increase the stiffness (18.34 MPa), compared to the control HA (12.60 MPa) sample. At the same time, the water-holding capacity of HA was tremendously increased (*p* < 0.05) by incorporating 5% Ge and the rest of the concentrations did not improve the water holding ability.

In general, the enzymatic degradation did not disintegrate the HA molecule significantly, since the HA is a ceramic-like mineral polymer. Similar to water solubility, there were no changes in the degradation pattern of HA, HA-1% Ge, and HA-3% Ge biomaterials throughout the study, however, HA-5% Ge biomaterial had more resistance against in vitro degradation, compared to the HA sample. On 60 days of treatment, HA, HA-1% Ge, and HA-3% Ge biomaterials were degraded at most of 35%, which was 22% in HA-5% Ge biomaterial.

**Figure 2:**
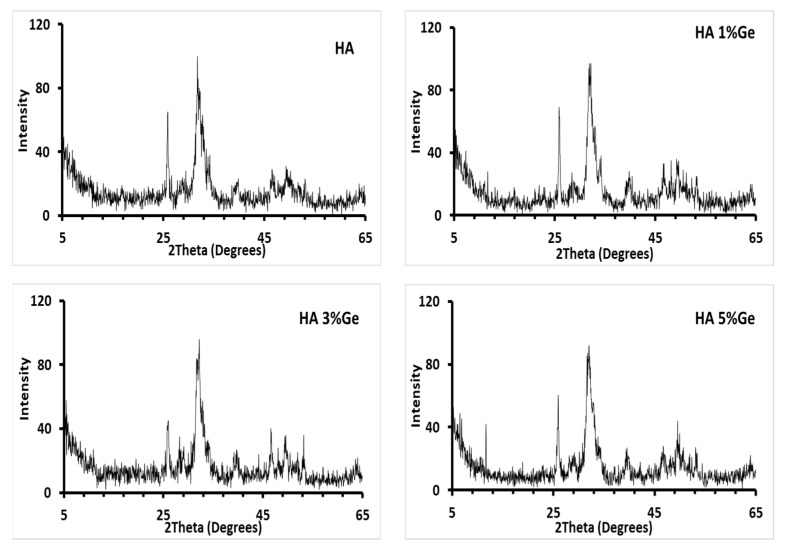
X-ray diffraction patterns of the composites HA-Ge biomaterials. HA-control, HA 1% Ge: HA with 1% germanium, HA 3% Ge: HA with 3% germanium, and HA 5% Ge: HA with 5% germanium.

Figure 2 shows the X-ray diffraction patterns of the composites HA-1% Ge, HA-3% Ge and HA-5% Ge biomaterials. The XRD patterns of the composite HA biomaterials showed peaks corresponding with HA (JCPDS standard data of HA, card no. 09-0432), regardless of the addition of germanium, due to the percentage of germanium not being enough to be detected with the XRD (below the limit of XRD detection). The granulometry data showed that the average particle size of the HA was 4.845 μm (result analysis report of particle size distribution test of HA and EDX of the Germanium are presented in the Supplementary Figures S3 and S4, respectively).

**Figure 3:**
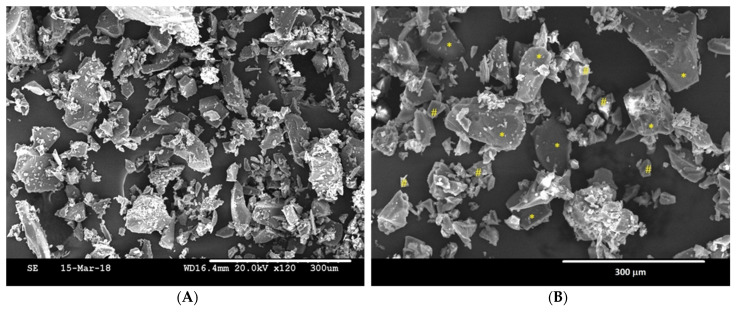
SEM images of the Ge (**A**) and HA-5% Ge powder composite (**B**). (*) HA particles and (#) Ge particles. Scale bar—300 µm.

Figure 3 shows the morphology of the HA-5%Ge composite before isostatic pressing as representative of all HA-Ge composites, since not much difference was observed in the surface of HA loaded with different concentrations. The composite was made up of particles of HA (*) with rounded edges, and the Ge particles (#) show edges more marked than HA and smaller in size, comparatively. EDX analysis showed that the obtained materials were stoichiometric HA with a Ca/P ratio of ~1.66, and the composites were a mixture of HA and Ge particles (Table 1).

### 3.2. Drug-Release

**Figure 4:**
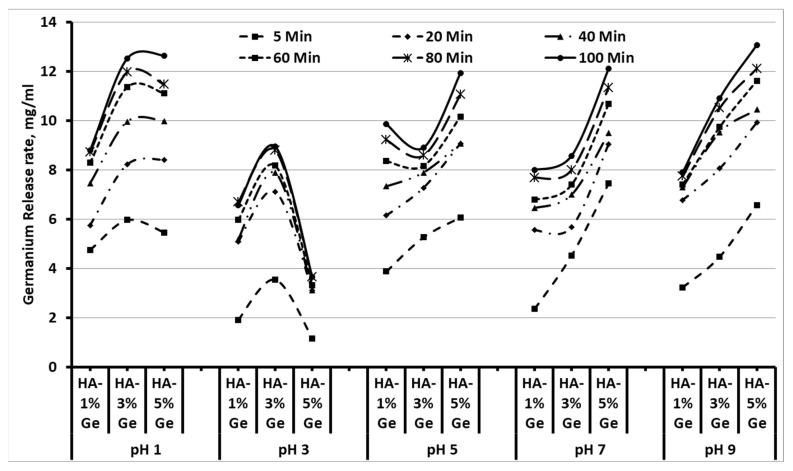
Drug-releasing pattern of HA-Ge biomaterials in different pH. HA-1% Ge-HA with 1% germanium, HA-3% Ge-HA with 3% germanium, and HA-5% Ge-HA with 5% germanium.

It is important to understand the releasing behavior of Ge by composite HA biomaterials to assess the biological behavior. Therefore, we tried to evaluate the Ge releasing behavior of composite HA in different mediums, such as SBF, DMEM and PBS, as well as in different pH at a period. As expected, the releasing rate of Ge was increased with respect to time and concentration in all the samples treated with different mediums and pH, however, the Ge-releasing pattern was different between test groups. For instance, the higher releasing rate of Ge was observed in HA-3% Ge at pH 1 and 3, and in HA-5% Ge at pH 5, 7 and 9, respectively (Figure 4).

**Figure 5:**
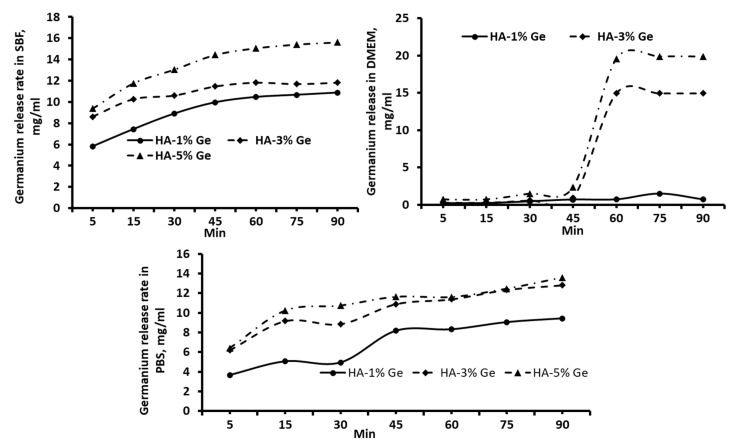
Drug-releasing pattern of HA-Ge biomaterials in different mediums. PBS—phosphate buffered saline, SBF—simulated body fluid, DMEM—DMEM culture medium. HA-1%Ge—HA with 1% germanium, HA-3%Ge—HA with 3% germanium and HA-5%Ge—HA with 5% germanium.

Among all samples tested, the maximum drug release was obtained in the HA-5% Ge sample at pH 7 and pH 9. Similar to pH, the releasing rate of Ge was steadily increased in all the test samples with respect to increasing duration. The release rate of Ge in PBS had a similar trend, compared to SBF, slowly releasing the Ge in medium up to 90 min, and not many changes were observed at 75 and 90 min. On contrary, there was a minimum amount of Ge released in DMEM medium until 45 min and a sudden release rate was noted at 60 min, and after that slightly decreased at 75 and 90 min by HA-3% Ge and HA-5% Ge. All these changes were not seen in HA-1% Ge, showing a slight increase rate of Ge in 75 min (Figure 5).

### 3.3. Contact Angle Test

**Figure 6:**
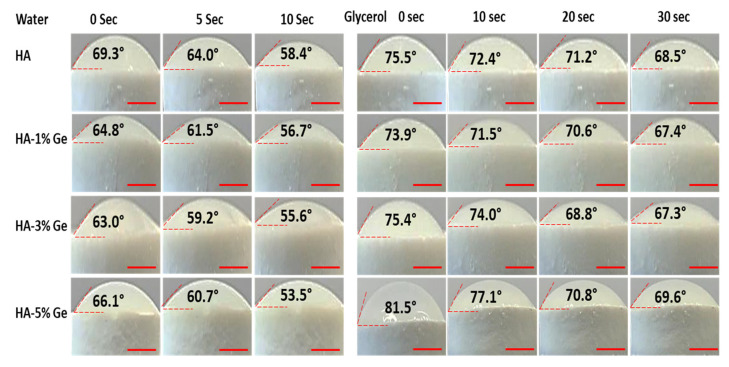
Contact angle behavior of HA-Ge biomaterials. Water (10 s) and glycerol (30 s) were used to test the hydrophilic and hydrophobic nature of HA-Ge composites. HA—control, HA-1%Ge—HA with 1% germanium, HA-3%Ge—HA with 3% germanium, and HA-5%Ge—HA with 5% germanium. Scale bar—0.4 cm.

The hydrophilic and hydrophobic behaviors of HA-Ge biomaterials were tested by using water and glycerol, respectively (Figure 6). As shown in Figure 5, the addition of Ge could increase the hydrophilic nature of HA and thereby reduce the water contact angle, and, after 15 s, the water contact angle of all HA samples became zero. At the same time, the hydrophobic nature of HA did not alter much by the incorporation of Ge, even though the glycerol contact angle was slightly increased in Ge-incorporated HA samples.

### 3.4. Protein Adsorption Ability

**Figure 7:**
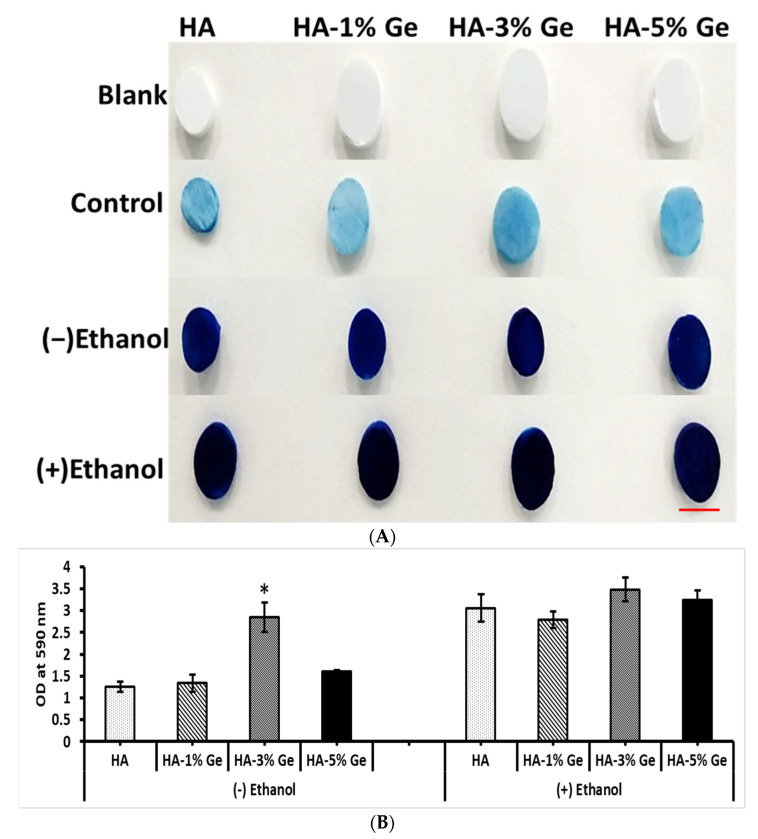
Protein absorption ability of HA-Ge biomaterials by CBB test. (**A**) CBB stained HA-Ge composites, Scale bar—1 cm, (**B**) Intensity of CBB staining in HA-Ge composites by UV absorption at 590 nm. Blank—samples without any treatment, control—FBS untreated samples with CBB stain, (−) Ethanol: HA-Ge composites without ethanol treatment and (+) Ethanol: HA-Ge composites with ethanol treatment. HA-control, HA-1%Ge-HA with 1% germanium, HA-3%Ge-HA with 3% germanium and HA-5%Ge-HA with 5% germanium. The UV absorption of control (2nd row in image (**A**)) was below the measurable range. * denotes statistical significance, *p* < 0.05 vs. HA.

The ability of HA-Ge samples in protein adsorption was evaluated using the CBB dye method. In general, all the samples were potentially bound to the serum protein and ethanol treatment could increase the ability of protein adsorption onto the HA-Ge surface. For instance, the quantification of protein by UV-Vis at 590 nm showed that the amount of protein absorbed more in ethanol-treated HA-Ge, compared to ethanol non-treated HA-Ge (Figure 7), however, no statistical significance was observed in protein absorption among ethanol-treated HA-Ge samples.

In contrast, non-treated HA-3% Ge had higher protein absorption (*p* < 0.05) than that of other non-treated HA-Ge samples and no statistical difference was observed between ethanol-treated and non-treated HA-3% Ge samples.

### 3.5. In-Vitro Bioactivity

**Figure 8:**
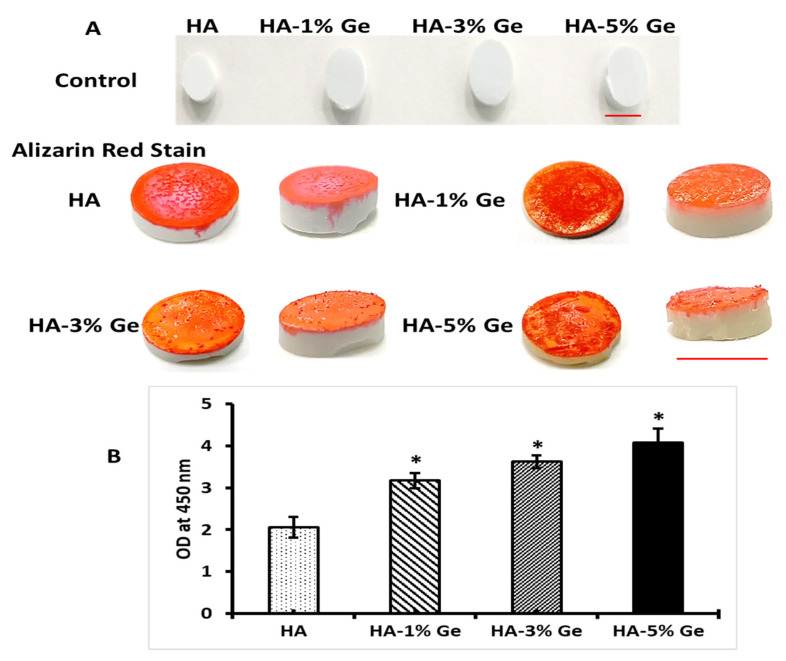
In vitro bioactivity of HA-Ge biomaterials after SBF treatment for 28 days with Alizarin red stain. (**A**)—samples with Alizarin red stain, Scale bar —1 cm, and (**B**)—UV absorption of Alizarin red stain on samples at 450 nm (**B**). HA-control, HA-1%Ge-HA with 1% germanium, HA-3%Ge-HA with 3% germanium and HA-5%Ge-HA with 5% germanium. The UV absorption of control (1st row in image (**A**)) was below the measurable range. The bioactivity of HA was significantly improved by Ge. * denotes statistical significance *p* < 0.05 vs. HA.

To evaluate the mineralization behavior to favor extracellular matrix formation, the HA-Ge samples were soaked in SBF for 30 days followed by quantification of mineral using alizarin red stain. The deposition of the apatite layer, which is mainly composed of calcium and phosphate ions, onto the HA-Ge surface was visualized by Alizarin red stain and the results showed that mineralization was favored by all the samples, however, the range of mineralization was directly proportional to the concentration of Ge. The formation of the apatite layer was increased by increasing Ge concentration and the level of apatite layer deposition was more in HA-5% Ge, compared to control HA (*p* < 0.05) (Figure 8). As shown in Figure 8, the characteristic crystal structure and morphology were seen in higher concentration of Ge (3 and 5%)-incorporated HA samples.

### 3.6. Effect of Ge on BM-MSCs

To optimize the Ge concentration and its effect on BM-MSCs proliferation, different concentration of Ge (0.001, 0.01, 0.1, 0.25, 0.5, 1, 2.5, 5 and 10 mg/mL) was chosen to culture BM-MSCs and evaluated by cell counting, H&E stain (Supplementary Figure S5) and fluorescence stains.

**Figure 9:**
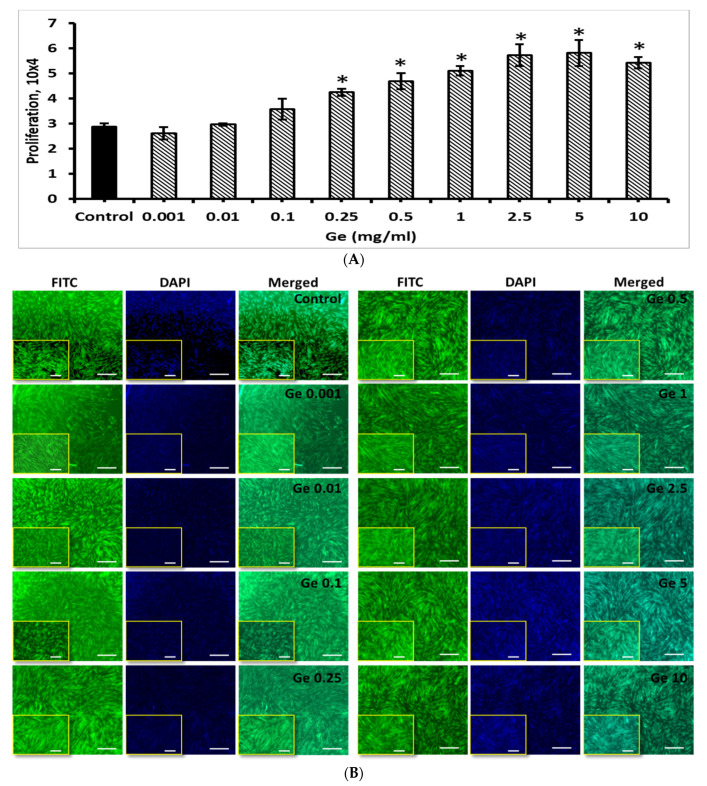
(**A**) Cell count and (**B**) Fluorescence (FITC, DAPI and merged) images of BM-MSCs cultured with Ge. Control—BM-MSCs cultured without Ge, Ge 0.001, 0.01, 0.1, 0.25, 0.5, 1.0, 2.5, 5 and 10 represent their respective concentration of Ge treated with BM-MSCs, respectively. Scale bar 5×-200 μm, 10×-100 μm (Insert) * denotes statistical significance, *p* < 0.05 vs. control.

As expected, the BM-MSCs growth was increased by increasing the concentration of Ge (Figure 9). Compared to control, no significant changes were observed in cells growth of BM-MSCs treated with 0.001–0.1 mg/mL Ge but increasing Ge concentration further from 0.25 to 10 mg/mL triggered BM-MSCs growth (*p* < 0.05). Though the BM-MSCs count increased steadily with the treatment of Ge with 1–10 mg/mL, they did not show any statistical significance among these groups.

### 3.7. Effect of HA on BM-MSCs

**Figure 10:**
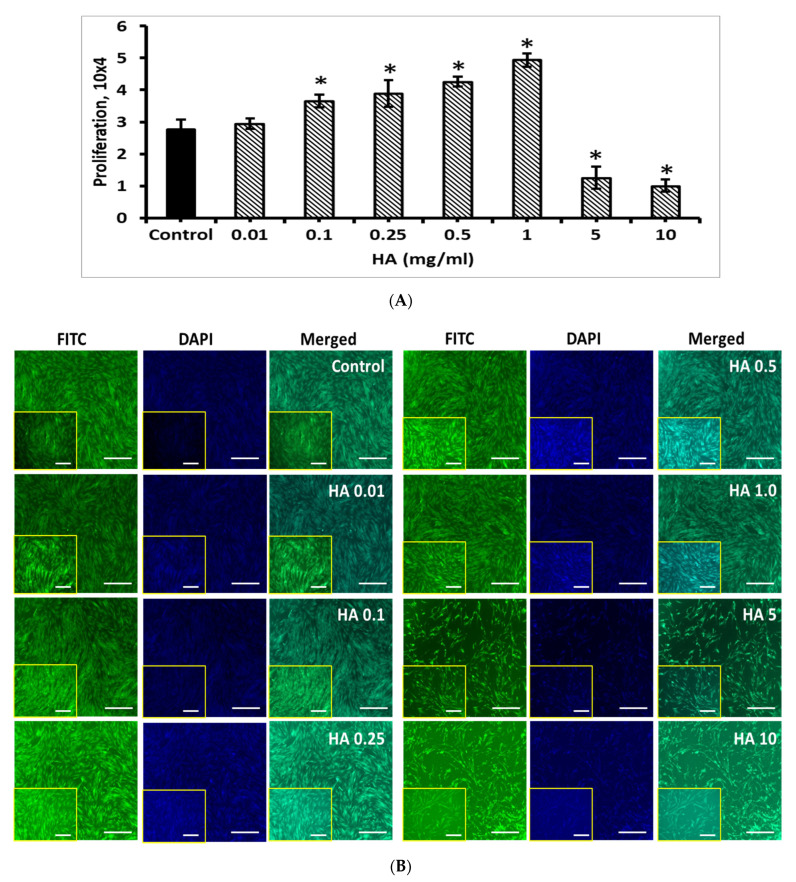
(**A**) Cell count and (**B**) Fluorescence (FITC, DAPI and merged) images of BM-MSCs cultured with HA. Control—BM-MSCs cultured without HA, HA 0.01, 0.1, 0.25, 0.5, 1.0, 5 and 10 represent their respective concentration of HA treated with BM-MSCs, respectively. Scale bar 5×-200 μm, 10×-100 μm (Insert). * denotes statistical significance, *p* < 0.05 vs. control.

Similar to Ge, the effect of HA on BM-MSCs were tested with different concentrations (0.01, 0.1, 0.25, 0.5, 1, 5 and 10 mg/mL). The rate of BM-MSCs proliferation was increased with increasing HA concentration from 0.01 to 1 mg/mL, compared to control, however, the higher concentration over 5 mg/mL significantly decreased the BM-MSCs proliferation (*p* < 0.05), which was clearly seen in FITC and DAPI fluorescence staining image (Figure 10). Interestingly, the BM-MSC proliferation was significantly improved by 1 mg/mL concentration of HA, compared to control BM-MSCs (*p* < 0.05).

### 3.8. Effect of HA-Ge Coating on BM-MSCs

**Figure 11:**
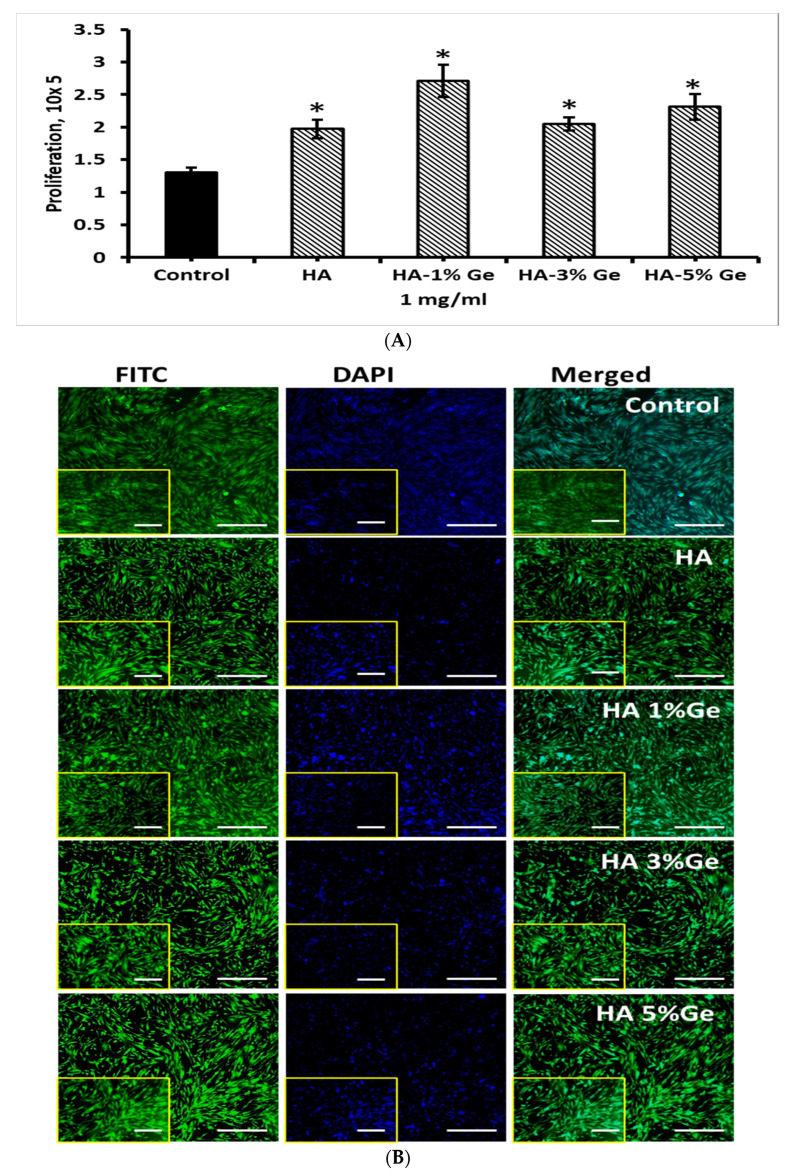
(**A**) Cell count and (**B**) Fluorescence (FITC, DAPI and merged) images of BM-MSCs cultured with HA-Ge coating. Control—BM-MSCs cultured without HA or HA-Ge composites, HA—HA without Ge, HA-1%Ge—HA with 1% germanium, HA-3%Ge—HA with 3% germanium, and HA-5%Ge—HA with 5% germanium. Scale bar 5×-200 μm, 10×-100 μm (Insert). * denotes statistical significance, *p* < 0.05 vs. control.

Based on the above findings, we investigated the coating efficiency of HA-Ge composites in BM-MSCs growth. For this purpose, the culture plates were coated with 1 mg/mL HA-Ge composites based on the results described in Section 3.6. As shown in Figure 11, the proliferation rate of BM-MSCs was increased with HA-Ge coatings, compared to control, and the cell growth was upregulated in BM-MSCs coated with HA-1% Ge composites, compared to other composites (*p* < 0.05) (the H&E staining of BM-MSCs on HA-Ge composites are shown in Supplementary Figure S6). Moreover, the effect was insignificant between BM-MSCs coated with HA and HA-3% Ge and HA-1% Ge and HA-5% Ge, respectively.

### 3.9. Cell Loading Density

**Figure 12:**
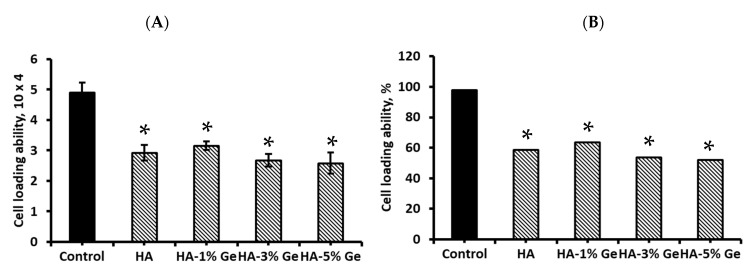
The total BM-MSCs seeding capacity of HA-Ge biomaterials. (**A**) and (**B**) are the actual and percentage of cell seeding density of BM-MSCs on HA-Ge composites, respectively. Control—BM-MSCs cultured without HA or HA-Ge composites, HA—HA without Ge, HA-1%Ge—HA with 1% germanium, HA-3%Ge—HA with 3% germanium, and HA-5%Ge—HA with 5% germanium. * denotes statistical significance, *p* < 0.05 vs. control.

The total capacity of HA-Ge biomaterial in BM-MSCs seeding result is presented in Figure 12. The total seeding density of BM-MSCs was 5 × 104 cells/sample and the total BM-MSCs attached to HA-Ge surface were 2.58–3.16 × 10^4^, which was significantly lower than control (without HA-Ge) BM-MSCs (4.89 × 10^4^) (*p* < 0.05). There was no significant change in cell loading density among HA and HA-Ge samples, which was further supported by H&E staining of HA-Ge-loaded BM-MSCs. The total percentage of cell loading ability was 97.8% in control and 51–63% in HA and HA-Ge samples, respectively.

## 4. Discussion

In general, HA is not soluble in water but soluble in gastric juice. In the 7 days experiment, the total percentage of HA solubility in all three mediums was about 1.9–2.3%, which was similar to the earlier works [42]. It was stated that the porosity and microstructure of HA affect the absorption of the solution [42,43]. However, the solubility of the HA sample was promoted by adding Ge. The physicochemical properties of Ge are believed to moderate the functional property of HA biomaterials. In general, the presence of two carboxyl groups in the molecular pattern of Ge makes it more hydrophilic, and, therefore, adding Ge into HA promotes the hydrophilic nature of HA-Ge biomaterials and thereby increases water solubility. Similarly, the stiffness of HA biomaterial was improved by the addition of Ge at 5%, however, lower concentration did not contribute to the stiffness of HA biomaterials. It was reported that the stiffness of HA was about 8 MPa to 520 MPa, which varies based on the source and fabrication method [44,45].

In the present study, the stiffness (compressive strength) of HA biomaterial was similar to the earlier work (5.67 to 7.66 MPa) using kaolin-reinforced hydroxyapatite scaffolds for bone regeneration [46]. However, high stiffness of about 400–900 MPa was reported for naturally derived hydroxyapatite scaffolds for bone biomaterials [47]. Similar to water solubility, the water holding capacity of HA was improved by Ge incorporation at a higher concentration (5%); however, lower concentration did not contribute any effect to water holding capacity. Similar to the present finding, the water holding ability of HA material was improved by the addition of biomolecules, such as hyaluronic acid [48] and cellulose nanocomposites [49].

From the in vitro biodegradation, it was clearly seen that the HA material was resistant enough to proteolytic digestion and very slow degradation was achieved throughout the study conducted for 60 days. The rate of degradation slowly increased with the duration of incubation. It is well known that the increasing incubation times are directly linear to the weight loss and degradation rate of the polymers during the in vitro degradation study [50].

The maximum degradation of the HA sample was observed at about 35% after 60 days of enzymatic digestion. Various factors, such as hydrophilic, pores interconnectivity, pore size, additives crosslinking and susceptible enzymes, regulate the degradation process of biomaterial [42], which is an important factor for cytotoxicity, i.e., inhibit or stimulate the metabolism of stem cells [51]. Previously, Ganesan et al. [42] investigated the in-vitro degradation of HA in PBS at 37 °C for 28 days and reported the degradation of around 1.2–1.75% at end of the experiment.

It has been reported that soaking the materials in solutions with an appropriate pH similar to the blood pH is the original simulation of the biomaterials being placed into the human body [43]. Therefore, we used SBF as a suitable medium to understand the actual drug-releasing behavior of HA biomaterials and the drug-releasing effect was compared with PBS and DMEM. Our results proved that the composite biomaterials could release Ge favorably with DMEM medium, which gives an additional impact of this material for stem cell culture. In addition, the optimum pH range for better Ge drug release of HA biomaterial was 7–9.

From the contact angle test, we further confirm that increasing Ge concentration from 1 to 5% could improve the hydrophilic and reduce the hydrophobic nature of HA. To support this finding, Zhang et al. recently reported that the hydrophilic surface of ZnO nanorod film was improved by combining it with germanium particles [52].

The inside-out signaling and cellular properties, such as proliferation, migration and differentiation of cells, are highly influenced by the interaction of extracellular matrix proteins, including vitronectin, laminin and fibronectin [53,54,55]. Hence, we evaluated the ability of HA-Ge biomaterials to absorb functional protein by CBB test. Moreover, the empirical evidence confirms the potential ability of ethanol in surface modification of polymer. Therefore, in this study, we compared the ability of HA-Ge biomaterials in functional protein adhesion with and without ethanol treatment.

The high protein absorption ability of HA, as evidenced by the CBB test, proves that it is a potential carrier system for protein drug delivery. For instance, Ho et al. developed a biodegradable polymer system using poly(lactic glycolic acid) (PLGA) and HA for bovine serum albumin (BSA) protein-controlled release system and concluded that this system could effectively carry the hydrophilic drug for sustained substance release [56]. Most importantly, the higher protein adhesion behavior of HA-Ge biomaterials ultimately evidenced that the HA-Ge biomaterials could enhance the cellular functionality of stem cells and support osteoblast differentiation. To support this finding, Tripathi et al. already claimed that the HA scaffold with good protein binding ability could potentially support the proliferation and differentiation of human osteoblast SaOS2 cells [57]. In the present study, the in vitro bioactivity results also proved the higher apatite layer deposition on HA-5% Ge biomaterials surface, which eventually supports the ability of HA-Ge biomaterials on extracellular matrix formation during osteogenesis.

Further, the effect of Ge on BM-MSCs proliferation was investigated in the present study. Our results disclosed that the BM-MSCs growth was potentially stimulated by Ge treatment, especially at 0.5–5 mg/mL. Interestingly, no cytotoxic effect of Ge was observed in all concentrations tested up to 10 mg/mL and BM-MSCs remain in their original shape with integrity DNA even after being treated with Ge, as evidenced by FITC and DAPI staining. To support the present finding, an earlier study conducted by Choi et al. by treating BM-MSCs with 50, 70 and 100 µg/mL germanium-enriched *Cordyceps militaris* (CMGe) disclosed that the proliferation of BM-MSCs was increased to 1.8 fold by 50 µg/mL CMGe than control cells.

Though the proliferation rate of BM-MSCs was upregulated by the treatment of HA up to 1 mg/mL, the effect was downregulated in a higher concentration of HA around 5 and 10 mg/mL. Therefore, 1 mg/mL of HA was chosen as an optimum concentration for the following coating experiments of BM-MSCs. The results showed that the wells coated with HA-1% Ge showed a favorable effect on BM-MSCs growth, compared to others. The exact reason for this effect is still unknown and needs further extensive research to understand the mechanism of HA-Ge in BM-MSCs.

From the available literature, the average percentage of viability using human stem cells for HA is reported to be around 70–100% [58,59,60]. Kumar et al. performed a cytotoxicity test on hydroxyapatite using mouse fibroblast 3T3-L1 cells with variable observation times [61]. Horta et al. investigated eggshell-based hydroxyapatite cells with 98.9% viability that were cultured in dental pulp stem cells (DPSCs) for 24 h [51]. The cytotoxicity of HA was tested with various type of cells, such as amniotic mesenchymal stem cell [62], bone-marrow-derived mesenchymal stem cells [63], adipose-derived mesenchymal stem cells [64], dental pulp stem cells [65], mouse fibroblast (l929 cell line) [19,42], kidney epithelial cells [16], bone cells [17,58,59,66], human lung fibroblast (mrc5) cells [18], human osteosarcoma cells [67], RAW cells [68] and L6 and MG63 cells [69].

The cell-loading ability of HA-Ge biomaterials was much lower than the control (without biomaterials) groups, which might be due to the lower surface area of HA-Ge biomaterials, compared to 2D culture plates. The loading density of BM-MSCs on polymer surfaces differs based on various factors, such as affinity, surface area, smoothness, stiffness and porosity [70,71].

## 5. Conclusions

In the present study, the physicochemical, functional and cytotoxic properties of HA biomaterials were tested with varying Ge concentrations. The HA biomaterials were synthesized with a particle size of 4.8 µm. The results concluded that the physicochemical and mechanical properties were improved by 5% Ge and the addition of Ge could improve the hydrophilic nature of HA biomaterials and biodegradation rate. The drug releasing rate of HA-Ge biomaterials was high in pH 7–9 and DMEM medium. Even though 5% Ge increased the physicochemical, mechanical and functional properties of HA biomaterials, the optimum percentage of BM-MSCs growth was noted as 1% Ge. Interestingly, the higher apatite layer deposition and protein absorption behavior ultimately proved the efficiency of HA-Ge biomaterials in bone cell differentiation. However, further studies are essential to prove their effects on osteogenesis using BM-MSCs with and without osteogenic inducers.

## Figures and Tables

**Table 1:** EDX microanalysis of the HA-Ge composites.

	Ca (atomic%)	P (atomic%)	Ge (atomic%)
HA	62.41	37.60	-
HA-1%Ge	61.78	37.22	1.01
HA-3%Ge	60.71	36.35	3.02
HA-5%Ge	59.3	35.72	5.01

HA-control, HA-1%Ge- HA with 1% germanium, HA- 3%Ge- HA with 3% germanium, and HA-5%Ge- HA with 5% germanium.

## Data Availability

Not applicable.

## Supplementary Materials

The following supporting information can be downloaded at: https://www.mdpi.com/article/10.3390/cells11192993/s1, Figure S1: Spectral absorption of Germanium powder. Maximum absorption at 215 nm; Figure S2: Standard curve obtained from different concentrations of germanium; Figure S3: The average particle size of HA by particle size distribution test using Granulometry. Figure S4: The EDX of the Germanium; Figure S5: H&E staining of BM-MSCs treated with 0.001–10 mg/mL Ge (A), 0.01–10 mg/mL HA (B) and HA-Ge composites (C). HA-control without germanium, HA-1%Ge-HA with 1% germanium, HA-3%Ge-HA with 3% germanium, and HA-5%Ge-HA with 5% germanium; Figure S6: H&E staining of HA-Ge composites with BM-MSCs. HA-control without germanium, HA-1%Ge-HA with 1% germanium, HA-3%Ge-HA with 3% germanium, and HA-5%Ge-HA with 5% germanium.
