# Supplementary material for: The Effect of Germanium-Loaded Hydroxyapatite Biomaterials on Bone Marrow Mesenchymal Stem Cells Growth
Source: Cells. 2022 Sep 26;11(19):2993. doi: 10.3390/cells11192993 (PMC9563598; doi:10.3390/cells11192993)
Supplement: Supplementary file 1 [file cells-11-02993-s001.zip › cells-1844568-supplementary.pdf]

The Effect of Germanium Loaded Hydroxyapatite Biomaterials on Bone Marrow Mesenchymal Stem Cells Growth

Jeevithan Elango [1,*], Rodion Bushin[1], Artiom Lijnev[1], Piedad Nieves De Aza[2], Carlos Pérez-Albacete Martínez[3], José Manuel Granero Marín[4], Ana Belen Hernandez[5], Luis Ramón Meseguer Olmo[5] and José Eduardo Maté Sánchez De Val[1,*]

[1] Department of Biomaterials Engineering, Faculty of Health Sciences, UCAM-Universidad Católica San Antonio de Murcia, Guadalupe, 30107 Murcia, Spain

[2] Instituto de Bioingeniería, Universidad Miguel Hernández, Avda. Ferrocarril S/n, Elche, Alicante, 03202, Spain

[4] Department of Implant Dentistry, Faculty of Medicine and Dentistry, UCAM-Universidad Católica San Antonio de Murcia, Murcia 30107, Spain

[5] Tissue Regeneration and Repair Group, Biomaterials and Tissue Engineering, Faculty of Health Sciences, UCAM-Universidad Católica San Antonio de Murcia, Guadalupe, 30107 Murcia, Spain

* Correspondence: srijeevithan@gmail.com and jelango@ucam.edu (J.E); jemate@ucam.edu (J.E.M.S.V)

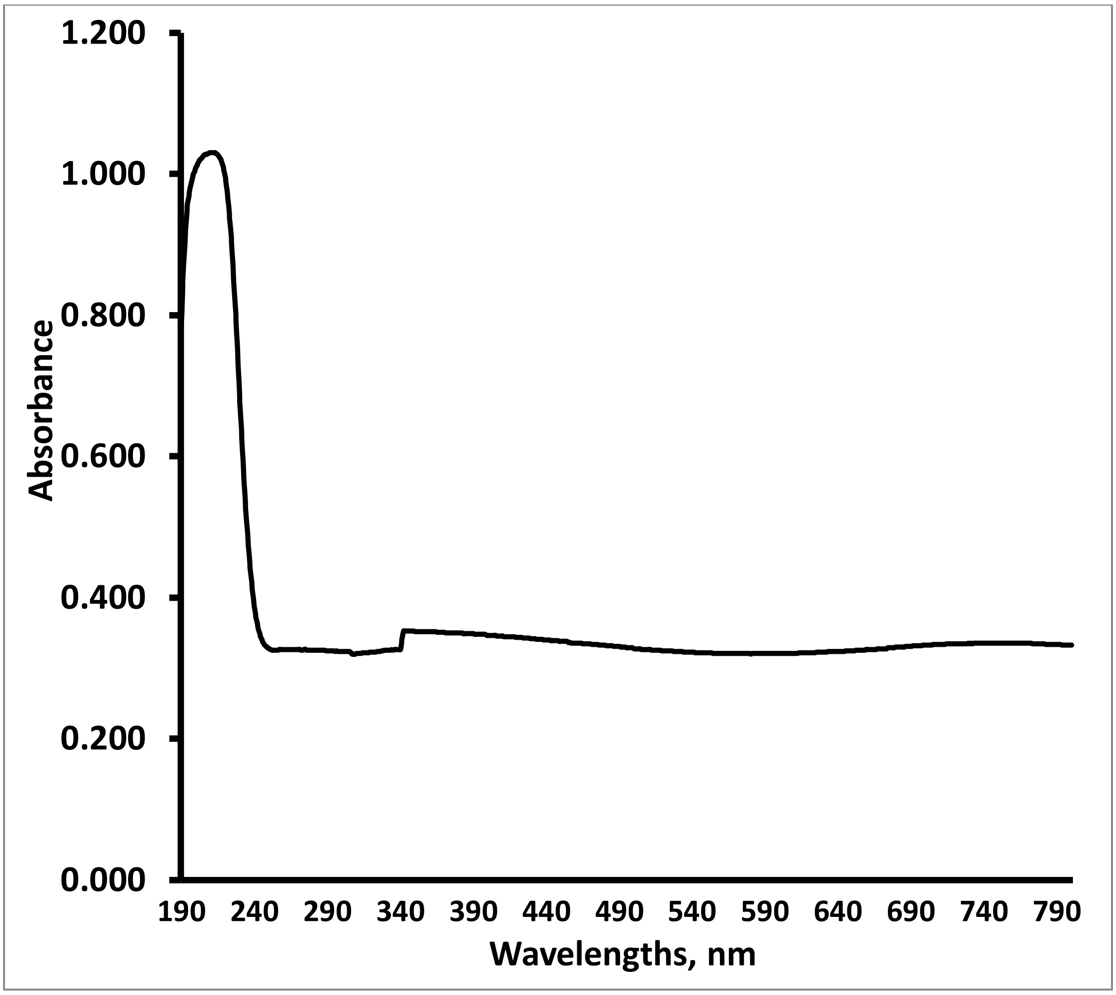

Figure S1. Spectral absorption of Germanium powder. Maximum absorption at 215 nm.

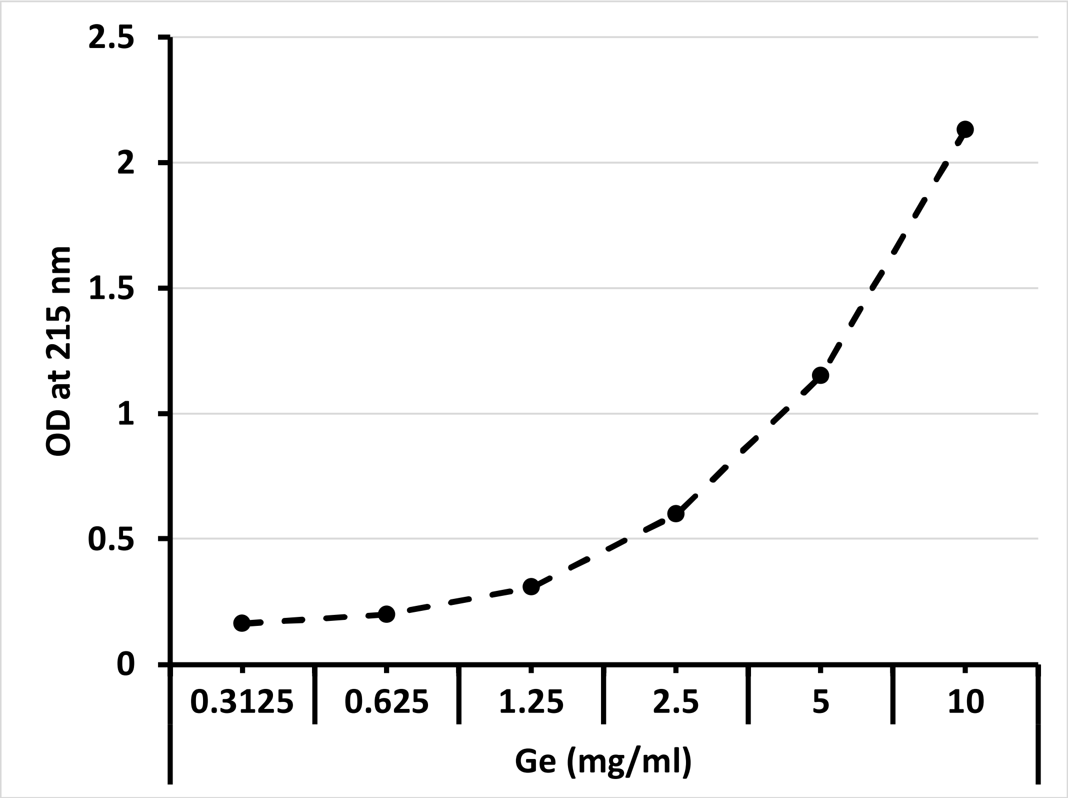

Figure S2. Standard curve obtained from different concentration of germanium

Instituto de Bioingeniería
Universidad Miguel Hernández

Result Analysis Report

Sample Name:
HA Piedad water - Average

SOP Name:
Hydroxyapatite

Measured:
miércoles, 28 de julio de 2021 11:39:30

Sample Source & type:
Factory = Paris

Measured by:
pavelasquez

Analysed:
miércoles, 28 de julio de 2021 11:39:31

Sample bulk lot ref:
123-ABC

Result Source:
Averaged

Particle Name:
Hydroxyapatite

Accessory Name:
Hydro 2000SM (A)

Analysis model:
General purpose

Sensitivity:
Enhanced

Particle RI:
1.650

Absorption:
0.01

Size range:
0.020 to 2000.000 um

Obscuration:
12.77 %

Dispersant Name:
Water

Dispersant RI:
1.330

Weighted Residual:
1.540 %

Result Emulation:
Off

Concentration:
0.0067 %Vol

Span :
1.720

Uniformity:
0.568

Result units:
Volume

Specific Surface Area:
1.48 m^2/g

Surface Weighted Mean D[3,2]:
4.063 um

Vol. Weighted Mean D[4,3]:
5.904 um

d(0.1): 2.210 um **d(0.5):** 4.845 um **d(0.9):** 10.546 um

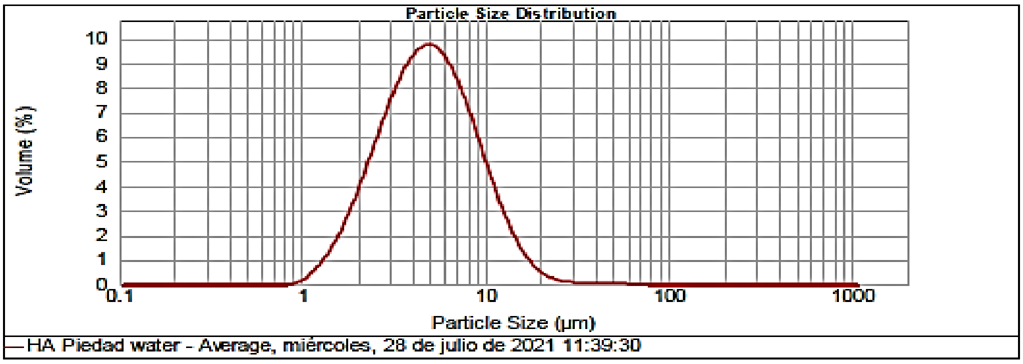

Size (μm)	Volume In %
0.020	0.00
0.022	0.00
0.025	0.00
0.028	0.00
0.032	0.00
0.036	0.00
0.040	0.00
0.045	0.00
0.050	0.00
0.056	0.00
0.063	0.00
0.071	0.00
0.080	0.00
0.089	0.00
0.100	0.00
0.112	0.00
0.126	0.00
0.142	

Size (μm)	Volume In %
0.142	0.00
0.159	0.00
0.178	0.00
0.200	0.00
0.224	0.00
0.252	0.00
0.283	0.00
0.317	0.00
0.356	0.00
0.399	0.00
0.448	0.00
0.502	0.00
0.564	0.00
0.632	0.00
0.710	0.00
0.796	0.00
0.893	0.09
1.002	

Size (μm)	Volume In %
1.002	0.23
1.125	0.51
1.262	0.83
1.416	1.27
1.589	1.83
1.783	2.48
2.000	3.22
2.244	4.00
2.518	4.79
2.825	5.55
3.170	6.23
3.557	6.79
3.991	7.17
4.477	7.34
5.024	7.30
5.637	7.02
6.325	6.54
7.096	

Size (μm)	Volume In %
7.096	5.88
7.962	5.09
8.934	4.25
10.024	3.39
11.247	2.59
12.619	1.88
14.159	1.30
15.887	0.85
17.825	0.52
20.000	0.31
22.440	0.18
25.179	0.11
28.251	0.08
31.698	0.07
35.566	0.07
39.905	0.07
44.774	0.07
50.238	

Size (μm)	Volume In %
50.238	0.06
56.368	0.04
63.246	0.01
70.963	0.00
79.621	0.00
89.337	0.00
100.237	0.00
112.468	0.00
126.191	0.00
141.589	0.00
158.866	0.00
178.250	0.00
200.000	0.00
224.404	0.00
251.785	0.00
282.508	0.00
316.979	0.00
355.656	

Size (μm)	Volume In %
355.656	0.00
399.052	0.00
447.744	0.00
502.377	0.00
563.677	0.00
632.456	0.00
709.627	0.00
796.214	0.00
893.367	0.00
1002.374	0.00
1124.683	0.00
1261.915	0.00
1415.892	0.00
1588.656	0.00
1752.502	0.00
2000.000	

Operator notes:

Malvern Instruments Ltd.
Malvern, UK

Mastersizer 2000 E Ver. 5.20
Serial Number : MAL1013724

File name: Ha Piedad Water
Record Number: 4

Figure S3. The average particle size of HA by particle size distribution test using Granulometry

Project 1 15/03/2018 11:32:34

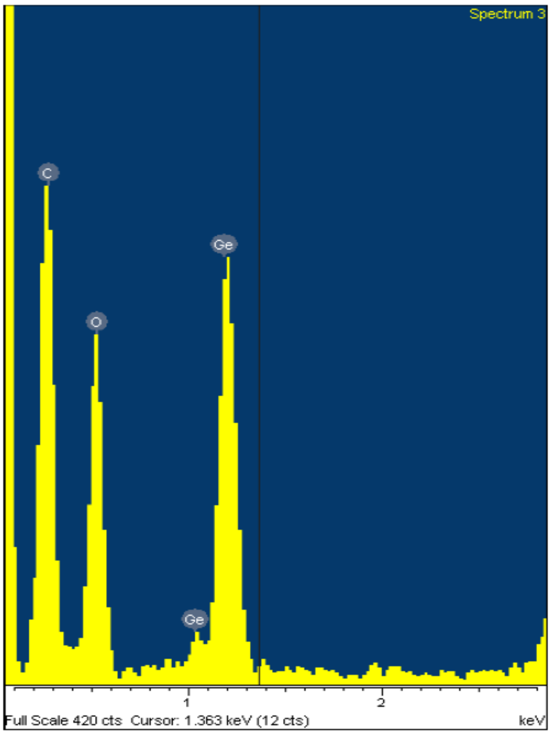

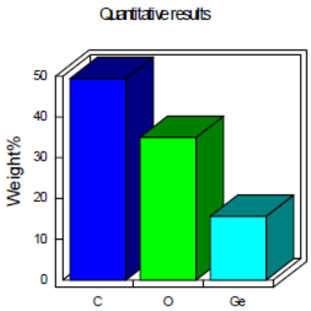

Processing option : All elements analyzed (Normalised)

Spectrum	C	O	Ge	Pd	Total
Spectrum 1	44.48	30.54	24.98		100.00
Spectrum 2	50.43	33.41	14.20	1.96	100.00
Spectrum 3	49.39	34.99	15.62		100.00
Max.	50.43	34.99	24.98	1.96	
Min.	44.48	30.54	14.20	1.96	

All results in Weight Percent

INCA

Figure S4. The EDX of the Germanium

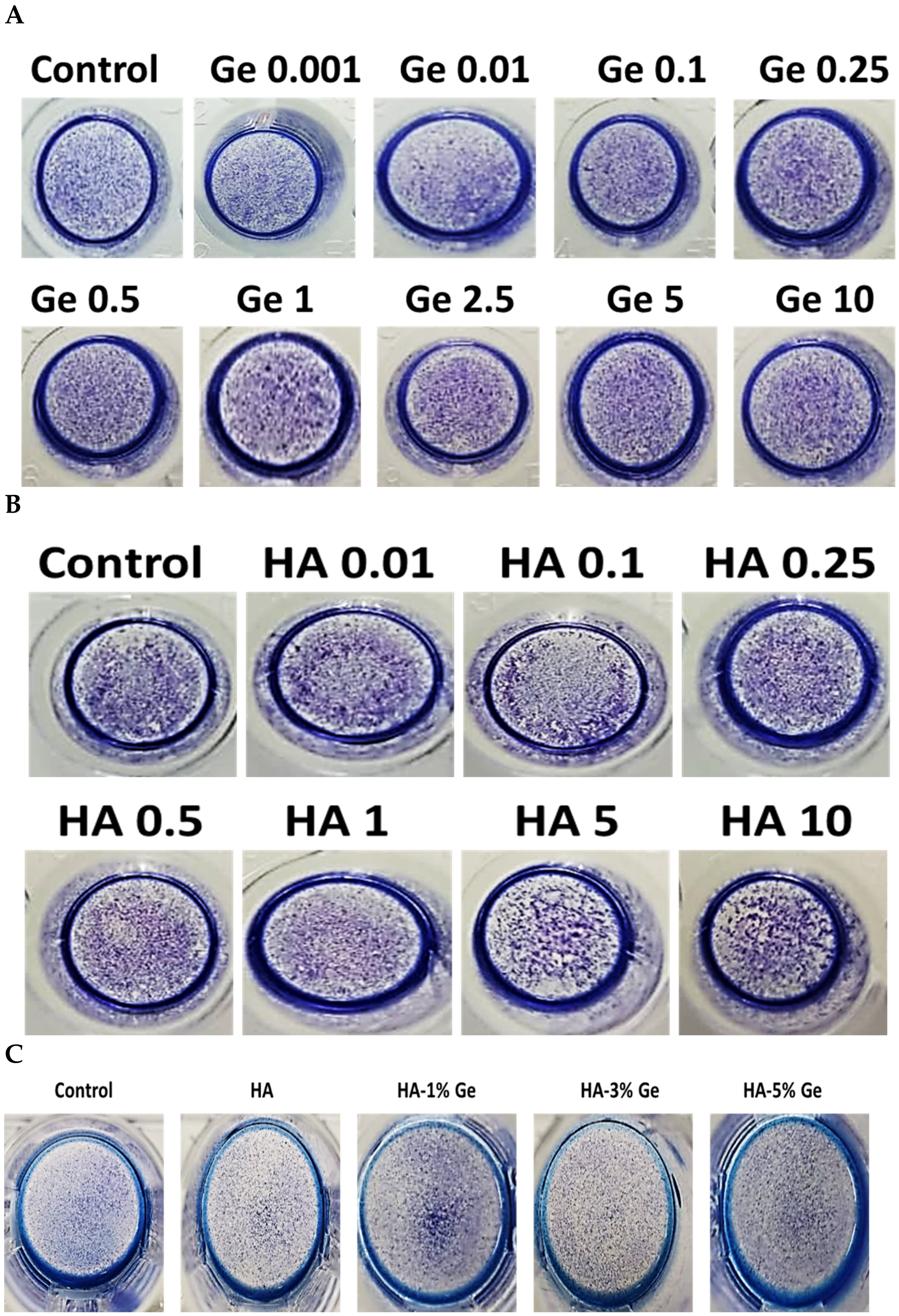

Figure S5. H&E staining of MSC cells treated with 0.001-10 mg/ml Ge (A), 0.01-10 mg/ml HA (B) and HA-Ge composites (C). HA-control without germanium, HA-1%Ge-HA with 1% germanium, HA-3%Ge-HA with 3% germanium, and HA-5%Ge-HA with 5% germanium

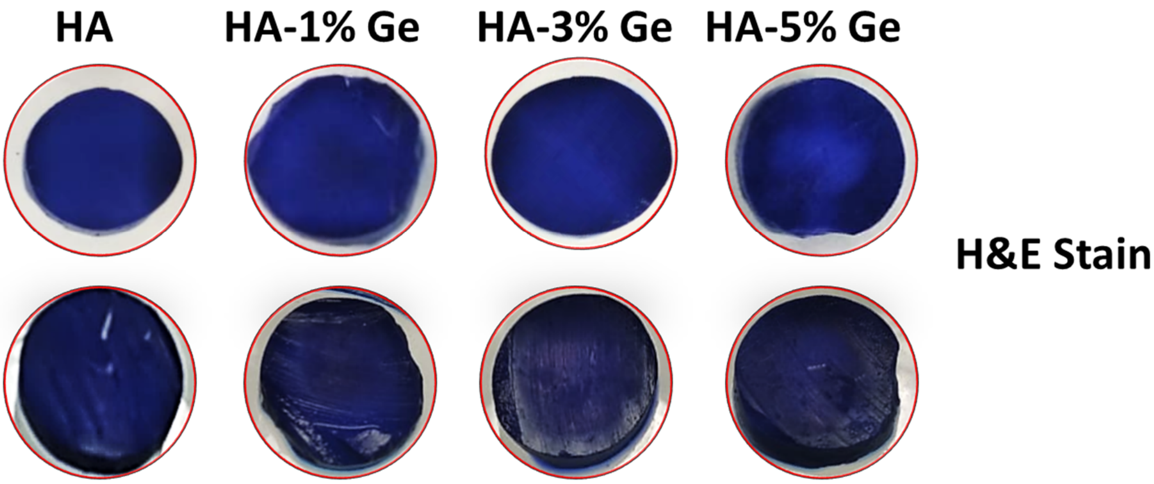

Figure S6 H&E staining of HA-Ge composites with MSC cells. HA-control without germanium, HA-1%Ge-HA with 1% germanium, HA-3%Ge-HA with 3% germanium, and HA-5%Ge-HA with 5% germanium
